# Pt nanoparticles decorated rose-like Bi_2_O_2_CO_3_ configurations for efficient photocatalytic removal of water organic pollutants†

**DOI:** 10.1039/c7ra12236e

**Published:** 2018-01-03

**Authors:** Huijuan Chen, Zhongfu Zhou, G. Neville Greaves, Salma Nigar, Huaqiang Cao, Tingkai Zhao, Xionggang Lu

**Affiliations:** School of Material Science and Engineering, Shanghai University Shanghai 200444 PR China; State Key Laboratory of Advanced Special Steel, Shanghai University Shanghai 200072 PR China z.zhou@shu.edu.cn; Key Laboratory of Material Microstructures, Shanghai University Shanghai 200444 PR China; Department of Physics, Aberystwyth University Aberystwyth SY23 3BZ UK; State Key Laboratory of Silicate Materials for Architectures, Wuhan University of Technology Wuhan 430070 China; Department of Materials Science and Metallurgy, University of Cambridge Charles Babbage Road Cambridge CB2 3QZ UK; Department of Chemistry, Tsinghua University Beijing 100084 PR China; State Key Laboratory of Solidification Processing, School of Materials Science and Engineering, Northwestern Polytechnical University Xi'an 710072 PR China

## Abstract

Pt nanoparticles decorated with rose-like Bi_2_O_2_CO_3_ configurations were synthesized *via* a simple photoreduction method at room temperature. The structure, morphology, optical and electronic properties, and photocatalytic performance of the as-prepared materials were characterized. Compared to pure Bi_2_O_2_CO_3_, the Pt/Bi_2_O_2_CO_3_ photocatalysts show better performance in decomposing RhB, BPA and OTC under visible light (*λ* > 420 nm). The enhanced photocatalytic activity of Pt/Bi_2_O_2_CO_3_ could be attributed to the modification in light absorption (*λ* > 420 nm) charge migration and the separation of photo-generated electrons (e^−^) and holes (h^+^). Free radical trapping experiments demonstrated that the main active species of the catalytic reaction are different in decomposing RhB and BPA.

## Abstract 2

Pt nanoparticles decorated with rose-like Bi_2_O_2_CO_3_ configurations were synthesized *via* a simple photoreduction method at room temperature.

## Graphical abstract

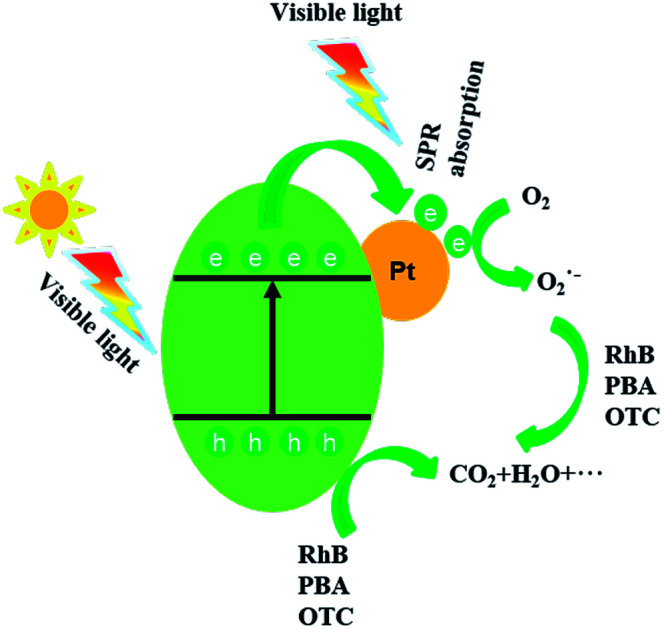

## 1. Introduction

Semiconductor-based photocatalysts, which can effectively absorb and utilize sunlight for the decomposition of pollutants, have aroused much attention recently.^1–5^ To date, many semiconductor photocatalysts have been developed for environmental purification and wastewater treatment, such as TiO_2_,^6,7^ ZnO,^8,9^ CuBi_2_O_4_,^10^ BiMoO_6_,^11^ BiOX (X = Cl, Br, I)^12–14^ and so on. As a newcomer of the aurivillius-related oxide family, Bi_2_O_2_CO_3_, which has an intergrowth of [Bi_2_O_2_]^2+^ layers and CO^3−^ layers,^15^ has been synthesized and used for the decomposition of rhodamine (RhB) in aqueous solution as a photocatalyst for the first time by zheng *et al.*^16^ Since then, a variety of methods have been developed to prepare Bi_2_O_2_CO_3_ catalysts with different morphologies, such as plate-like, flower-like and sponge-like configurations, with the corresponding band gap ranging from 3.5 eV to 2.87 eV (ref. 17).

For practical applications of photocatalysts, there is always a need to improve performance. Commonly, many researchers often construct heterojunctions with other materials to improve performance, such as MoS_2_/Bi_2_O_2_CO_3_,^18^ PPy/Bi_2_O_2_CO_3_,^19^ BiOBr/Bi_2_O_3_ (ref. 20) and so on. Recently, nanometer-sized noble metal particles have been deposited on the various photocatalysts with this aim.^21–24^ Besides, the surface plasmon resonance (SPR) effect of noble metal nanoparticles can enhance the visible light absorption of the catalysts.^25^ The system of noble nanoparticles decorated photocatalysts have been studied so for include: Pt/Bi_12_O_17_Cl_2_,^20^ Pt/TiO_2_,^26^ Au/WO_3_,^27^ AgCO_3_/Ag/WO_3_ (ref. 28) and Ag/Bi_2_O_2_CO_3_.^29^

In the present work, a new system has been developed consisting of Pt nanoparticles deposited on rose-like Bi_2_O_2_CO_3_*via* a simple photoreduction process. The material exhibits excellent photocatalytic activity compared with pure Bi_2_O_2_CO_3_ for the decomposition of RhB, under visible light irradiation. The catalyst also performs well in the degradation of colourless bisphenol A (BPA) and oxytetracycline (OTC).

## 2. Experimental

### 2.1 Materials

All of the reagents in this study were of analytical grade and used without further purification. Bismuth nitrate pentahydrate (Bi(NO_3_)_3_·5H_2_O), tri-sodium citrate (Na_3_C_6_H_5_O_7_) and chloroplatinic acid (H_2_PtCl_6_) were purchased from Sinopharm Chemical Reagent Co., Ltd. (Shanghai, China). Distilled water was used for the whole experiments.

### 2.2 Fabrication of rose-like Bi_2_O_2_CO_3_

Rose-like Bi_2_O_2_CO_3_ were synthesized following a previous recipe with slight modifications.^17^ Briefly, 0.4117 g bismuth nitrate pentahydrate and 0.3395 g tri-sodium citrate were added into 35 mL deionized water under vigorous stirring at room temperature. Then, the above solution was subjected to ultrasonic treatment (about 10 min) and followed by stirring for 3 h. Subsequently, the pH value of the milk white solution was adjusted to about 9 using diluted 28% (mass ratio) NH_3_·H_2_O solution. After about 2 h stirring, the final diaphanous solution was autoclaved at 180 °C for 30 h. After being cooled to room temperature, the precipitates were centrifuged and washed thoroughly abundant distilled water and ethanol, and fully dried at 60 °C in an oven to obtain rose-like Bi_2_O_2_CO_3_ (denoted BOC thereafter).

### 2.3 Fabrication of rose-like Pt/Bi_2_O_2_CO_3_

The platinization of Bi_2_O_2_CO_3_ were carried out following the typical photoreduction method.^30,31^ The surface of the microcrystals were platinized by photoreduction of Pt(iv) ions with UV light. In our experiments, 0.5 g Bi_2_O_2_CO_3_ powder was added into 100 mL deionized water, and then mixed with 4 g L^−1^ H_2_PtCl_6_ solution of different volumes under magnetic stirring. After 5 min ultrasonic treatment, the obtained suspension were poured into a 150 mL quartz photoreactor equipped with a water-cooled mercury immersion lamp. After 4 h irradiation, the products were washed several times by deionized water and then dried fully at 60 °C in an oven. The amount of H_2_PtCl_6_ solution was optimized to control the targeted Pt content in the photocatalysts respectively, 0.5 wt%, 1.0 wt%, 1.5 wt%, 3.0 wt%, 5.0 wt% and 10.0 wt% Pt/Bi_2_O_2_CO_3_. The corresponding samples are denoted as 0.5 P-BOC, 1.0 P-BOC, 1.5 P-BOC, 3.0 P-BOC, 5.0 P-BOC and 10.0 P-BOC.

### 2.4 Characterization

The properties of crystal phase of the samples were analyzed with Japan's Neo-Confucianism company (D\Max-2200) X-ray diffractometer (XRD, Cu Kα radiation at 40 kV and 40 mA in the 2*θ*, ranging from 10° to 70°) with a scanning rate of 4° per minute. The morphology and microstructure of the catalysts were characterized using a scanning electron microscope (SEM, JEOL, JSM-6700F) equipped with an energy-dispersive X-ray spectroscope (EDS) and high resolution transmission electron microscopy (HRTEM JEOL, JEM-2100F, with an accelerating voltage of 200 kV). The valence states of the samples were detected using X-ray photoelectron spectroscopy (XPS) on a Thermo ESCALAB 250 Xi with Al Kα, in which the XPS PEAK software was used for the fitting of the XPS. Nitrogen adsorption–desorption isotherms were collected on a Micromeritics-Gemini adsorption analyzer (ASAP, 2020M + C, USA) with all the samples degassed at 200 °C for 5 h before the measurements. The Brunauer–Emmett–Teller (BET) surface area was calculated from the linear part of the BET plot. UV-vis diffuse reflectance spectra (DRS) were recorded on a U-4150 spectrophotometer with BaSO_4_ as the background between 200 and 800 nm. Solid-state PL spectra were recorded on a Fluorescence spectrophotometer (PL, RF-5301, Japan).

### 2.5 Photocatalytic test

The photocatalytic activity of the obtained the P-BOC photocatalysts were evaluated by degradation RhB, BPA, and OTC. In a typical photocatalytic experiment, a 300 W Xe lamp with a UV-cut filter (*λ* > 420 nm) was used as the light source. 50 mg Pt/Bi_2_O_2_CO_3_ photocatalysts were added into 50 mL of RhB (40 mg L^−1^), BPA (10 mg L^−1^), and OTC (10 mg L^−1^) aqueous solution, respectively. The suspensions were sonicated for 2 min and then kept in the dark for 30 min before being irradiation with vigorous stirring to reach an adsorption–desorption equilibrium between photocatalysts and the organic dyes (see Fig. S1 in the ESI†). Then the solution was exposed to visible light irradiation under magnetic stirring and at a given time, 2 mL of sample was taken from the suspension and immediately centrifuged at 8000 rpm for 10 min. Then UV-vis spectra of the liquid supernatant were recorded using a UV-4100 spectrophotometer.

### 2.6 Photoelectrochemical measurements

The transient photocurrent responses were measured on the CHI760D electro-chemical work station with a standard three-electrode system. The working electrodes were prepared as follows: firstly, ITO glasses (1 cm × 1 cm) were ultrasonically cleaned in deionized water for about 10 min. Secondly, 80 mg the as-prepared photocatalysts, 10 mg acetylene black, and 10 mg poly(vinylidene difluoride) were mixed into a slurry and coated onto the 1 cm × 1 cm ITO glass. Finally, the working electrodes were dried in a vacuum oven at 60 °C for 12 h. A 300 W Xe lamp was used as light source with a UV-cut filter (*λ* > 420 nm). Na_2_SO_4_ aquous-solution (0.5 M) was utilized as the electrolyte for the photocurrent measurement.

## 3. Results and discussion

### 3.1 Phase structure of materials

**Fig. 1:**
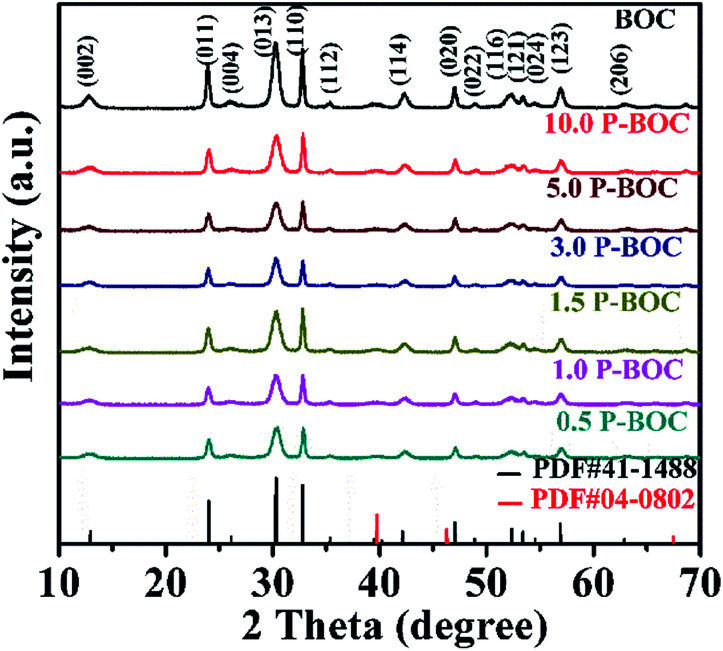
XRD patterns of BOC and the as-prepared P-BOC samples with different Pt content.

The phase analysis of seven samples with different Pt content were carried out using X-ray diffraction (XRD). Fig. 1 shows patterns of the as-prepared powders. From which it can be seen that they all correspond to the tetragonal phase of Bi_2_O_2_CO_3_, (*a* = *b* = 3.865 Å, *c* = 13.67 Å (JCPDS card no. 41-1488)). There are no obvious diffraction peaks of Pt crystallites observed for the samples of the P-BOC, which is attributed to the low concentration of Pt and Debye–Scherrer broadened due to their nano-crystalline dimensions, compared to the larger microcrystalline structure of the Bi_2_O_2_CO_3_ rosettes. Similar phenomena have been reported for metals in Pt/TiO_2_,^26^ Ag_2_O/Bi_2_O_2_CO_3_ (ref. 32) and Pt/Bi_12_O_17_Cl_12_.^25^

**Fig. 2:**
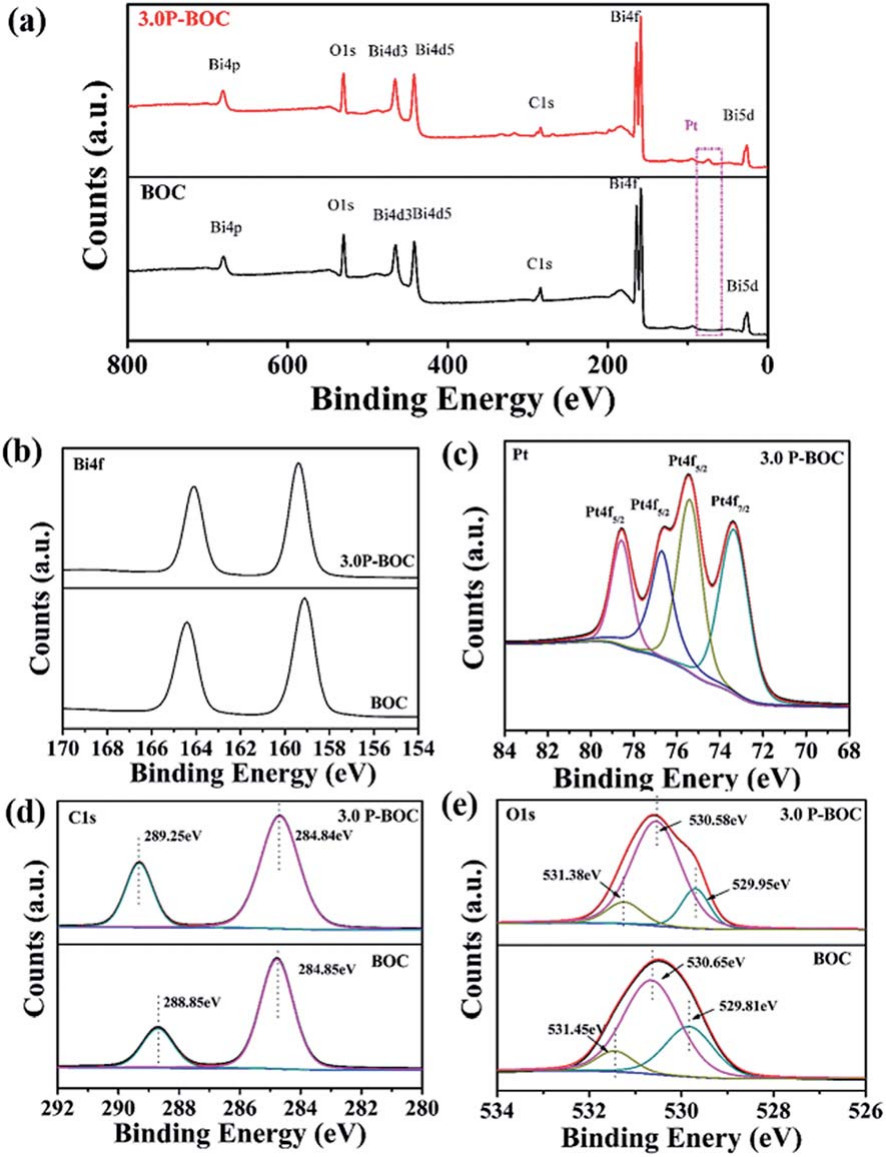
XPS survey spectra of BOC and 3.0 P-BOC: (a) survey spectra; (b) Bi 4f spectra; (c) Pt spectra; (d) C 1s spectra and (e) O 1s spectra.

X-ray photoelectron spectroscopy (XPS) was used to probe the electronic states within these photocatalysts, together with further information concerning the chemical composition. The XPS spectroscopy surveys of the electronic structure of BOC and 3.0 P-BOC are shown in Fig. 2. The existence of Pt in 3.0 P-BOC but not in BOC is confirmed by the relatively low intensity peak, present in the former but absent for the latter, and highlighted by the pink dot-line in Fig. 2(a). The high-resolution XPS spectra of Bi 4f, Pt 4f, C 1s and O 1s for 3.0 P-BOC and BOC samples are shown in Fig. 2(b–e). The peaks with binding energy at about 164.4 eV/164.22 eV and 159.1 eV/159.21 eV in both BOC and 3.0 P-BOC are associated with Bi 4f_5/2_ and Bi 4f_7/2_, respectively, indicating Bi is in the stable Bi^3+^ form (Fig. 2(b)).^33^ The high-resolution spectrum of Pt element in P-BOC (in Fig. 2(c)) shows four separate peaks with binding energy of 73.5 eV, 75.4 eV, 76.7 eV and 78.6 eV. The binding energies 73.5 eV, 75.4 eV and 78.6 eV correspond to the Pt 4f_7/2_ Pt 4f_5/2_ and Pt 4f_5/2_ states, respectively and are characteristic of metallic Pt^25^. By contrast the binding energy of 76.7 eV is associated with the Pt 4f_5/2_ state, indicating the existence of Pt(iv).^34^ It can be concluded that metallic Pt nanoparticles are present on the surface of P-BOC and that it is also likely that some residual Pt ions may also present within the sample. The binding energy of C 1s is 284.85 eV/284.84 eV and 289.85 eV/289.25 eV as shown in Fig. 4(d), which are in good agreement with the adventitious carbon expected from the instrument and O=C–O carbonate carbon in Bi_2_O_2_CO_3_, as reported in the literature.^35^ The O 1s spectra can be divided into three Gaussian–Lorentzian peaks (Fig. 2(e)). The 531.45 eV/531.38 eV, 530.65 eV/530.58 eV and 529.81 eV/529.95 eV features can also be assigned to the absorption of carbonate species and hydroxyl groups, characteristic of Bi_2_O_2_CO_3_ of Bi–O separately.^36^

**Fig. 3:**
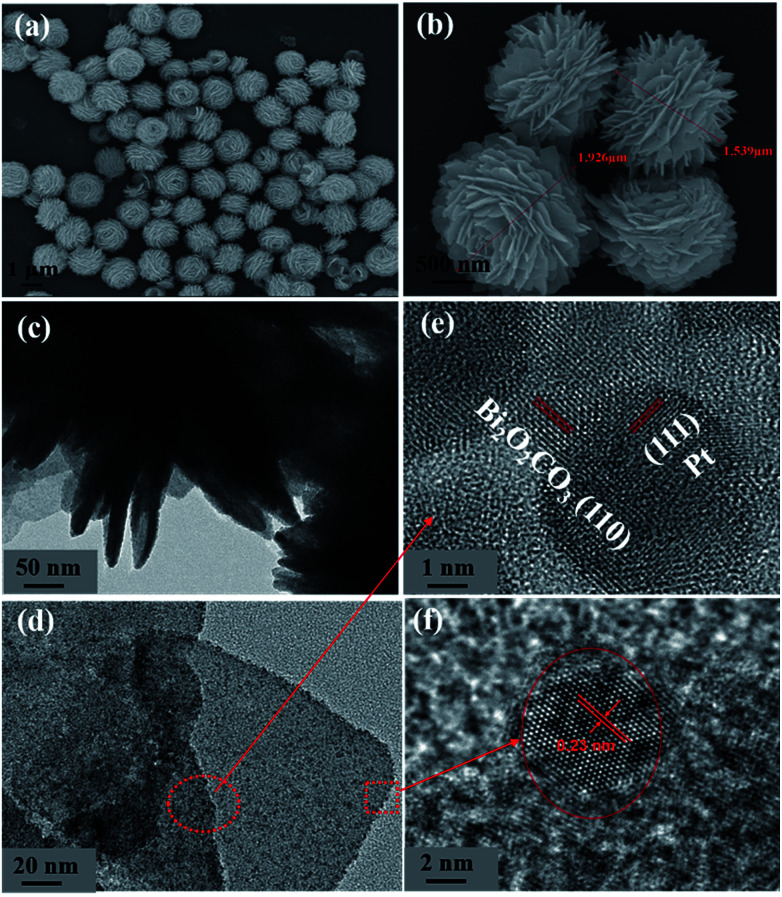
The characterization of morphologies of BOC and P-BOC. SEM images of BOC (a and b). TEM images (c and d) and HRTEM (e and f) of 3.0 P-BOC.

The morphologies of the BOC and the P-BOC samples are shown in the Fig. 3(a and b). From these SEM images, one can see that the BOC particles adopt a rose-like morphology about 1–2 μm in size, each being composed by leaf-like nano-sheets. These images are consistent with what has been reported previously.^17^ Again, in conjunction with XRD, there is no clear evidence of Pt phases on the SEM scale, even for the most concentrated 10.0 P-BOC, or where they might be situated.

**Fig. 4:**
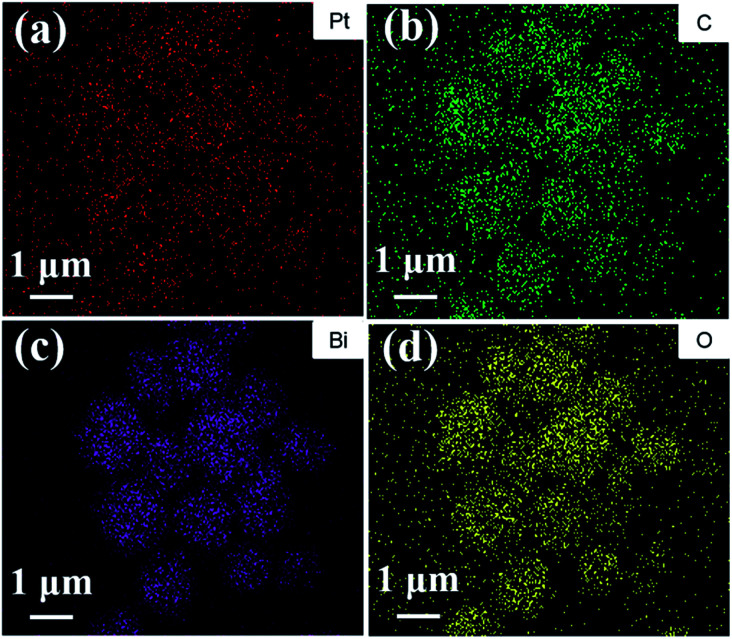
The elemental distribution maps for (a) Pt, (b) C, (c) Bi and (d) O of the as-prepared 3.0 P-BOC.

Fig. 3(c and d) show TEM images of the 3.0 P-BOC samples. Numerous nanometer sized particles can be seen on the nanosheets of the BOC particles. Fig. 3(e and f) show HRTEM images of the 3.0 P-BOC sample. These indeed show that the catalyst system is composed of Bi_2_O_2_CO_3_ nanosheets supporting Pt nanoparticles on the scale of several nanometers. Measurements of the lattice fringes further confirm that the phases shown in the figures correspond to Bi_2_O_2_CO_3_ nanosheets supporting Pt nanocrystals. In particular, the 0.27 nm of lattice spacing can be assigned to the (110) lattice planes of tetragonal structured Bi_2_O_2_CO_3_; while the 0.23 nm of lattice spacing is close to the separation of (111) lattice plane in crystalline Pt. The distribution of Pt was investigated by elemental mapping under SEM, as shown in Fig. 4(a–d). The results show that Pt element is well distributed in the particles and far less concentrated than the distributions of C, Bi and O. Furthermore, the whole Pt contents in the P-BOC samples were also measured by ICP-OES technology. The contents of Pt were calculated to be 0.48 at%, 0.91 at%, 1.39 at%, 2.59 at%, 4.15 at% and 6.17 at% in 0.5 P-BOC, 1.0 P-BOC, 1.5 P-BOC, 3.0 P-BOC, 5.0 P-BOC and 10.0 P-BOC.

**Fig. 5:**
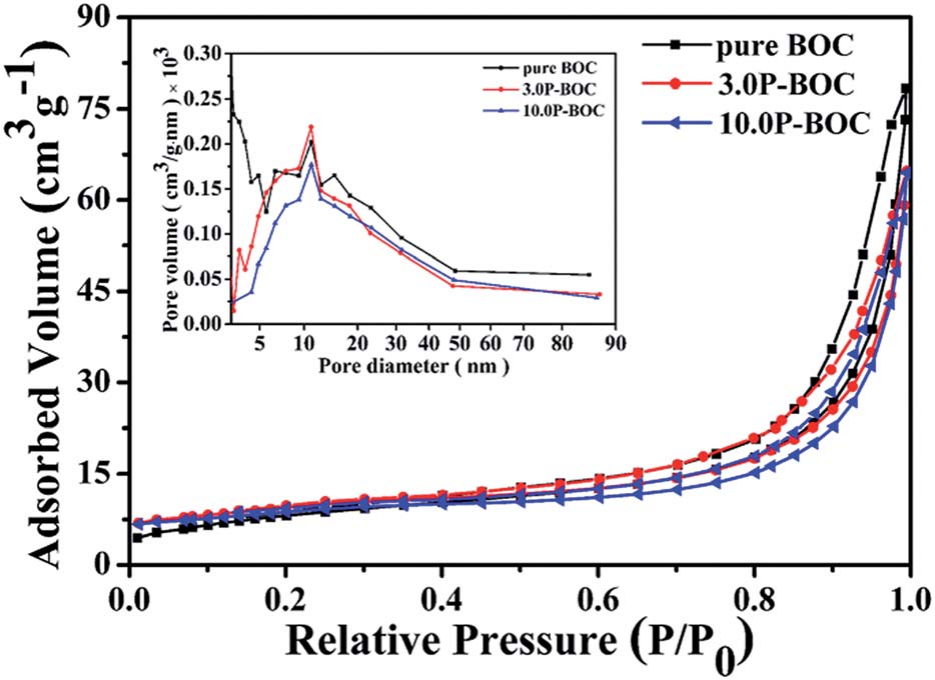
Nitrogen absorption–desorption isotherms for BOC, 3.0 P-BOC and 10.0 P-BOC. The inset shows the corresponding pore-size distribution curves.

The Brunauer–Emmett–Teller (BET) specific surface areas and pore architecture of the BOC and of P-BOC were investigated using N_2_ adsorption–desorption isotherms. As shown in Fig. 5 that the isotherms of all three samples, BOC, 3.0 P-BOC, and 10.0 P-BOC, are of type IV, and indicate the presence of mesopores. Furthermore, the shape of hysteresis loops is similar with that of the H3 hysteresis, which indicates the formation of slit-like pores.^37^ The derived pore size distributions of the three samples are shown in the inset of Fig. 5, showing that pores are nanometer sized, and also that the addition of Pt has little influence on the size distribution. The specific surface area of the three samples are calculated to be 29.6977 m^2^ g^−1^, 35.4543 m^2^ g^−1^, and 29.4472 m^2^ g^−1^, respectively for BOC, 3.0 P-BOC and 10.0 P-BOC.

### 3.2 Optical property of materials

**Fig. 6:**
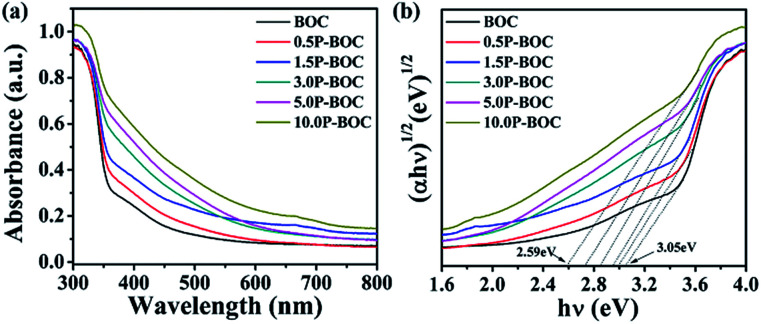
(a) UV-vis diffuse reflectance spectra and (b) band gap energies of different BOC's.

The UV-vis absorption spectra described in the Fig. 6(a) indicates that the absorption edge of the BOC is located at 400 nm. As the Pt content increases, the light absorption of the P-BOC is gradually enhanced, which can be attributed to the SPR of the Pt nanoparticles^38^ – the peaks becoming broader and stronger due to the Pt nanoparticle coverage on the surface of BOC. Based on the diffuse reflection spectra, the band gap energies *E*_g_ for different amounts of Pt can be determined according to^39^:*αhν* = *A*(*hν* − *E*_g_)*n*/2where *α*, *h*, and *ν* are the optical absorption coefficient, Planck's constant and the frequency of light. *A* is a coefficient and the exponential factor *n* relates to the type of optical transition equaling 1 for direct transitions and 4 if these are indirect. For Bi_2_O_2_CO_3_, the value of *n* is 1.^40^ The corresponding (*αhν*)^1/2^*vs. hν* curves for the samples are plotted in the Fig. 6(b), where the band gap energy can be seen to gradually increase with Pt content from 3.05 eV for the BOC to 2.59 eV for 10.0 P-BOC. This indicates that by adding Pt nanoparticles (Fig. 2(f)) the band gaps of BOC can be reduced, leading to a wider absorption capability. It is suggested that the Pt nanoparticles not only enhance the absorption of visible light owing to SPR, but also can change the energy equilibrium and reducing energy levels as a result of the Schottky barrier at the interface (Fig. 2(e and f)) between the BOC nanosheets and the Pt nanoparticles.^41^

### 3.3 Photocatalytic properties of materials

**Fig. 7:**
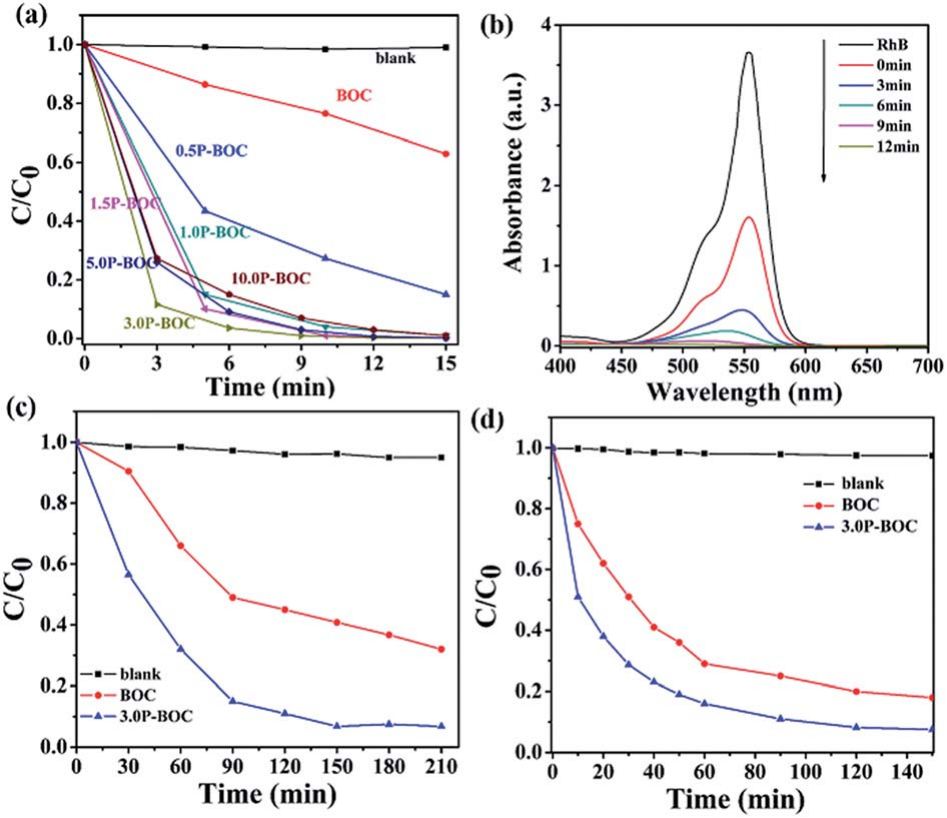
(a) Photocatalytic degradation of RhB (40 mg L^−1^) with the BOC and the P-BOC as photocatalysts under visible light irradiation (*λ* > 420 nm). (b) The temporal evolution of the absorption spectra of RhB in the presence of the 3.0 P-BOC samples under visible light irradiation. Photocatalytic degradation of (c) BPA (10 mg L^−1^) and (d) OTC (10 mg L^−1^) with the BOC and the 3.0 P-BOC as photocatalysts under visible light irradiation (*λ* > 420 nm).

The photocatalytic activity of BOC and P-BOC with different Pt loading were evaluated by observing the progressive degradation of RhB, BPA and OTC under visible light irradiation. Fig. 7(a and b) show the reduction of RhB concentration with irradiation time for different Pt loading, and the changes in the absorption spectra of RhB solution with 3.0 P-BOC as a function time. It is found that the photo-degradation of RhB can be ignored when the photocatalysts are absent. As illustrated in Fig. 7(a), the photocatalytic activity is enhanced with the addition of Pt nanoparticles. The 3.0 P-BOC exhibits the best performance where the degradation rate can reach 99.9% within 12 min. Further increasing the content of Pt reduce the increase in photocatalytic activity, which may due to further addition of Pt obscuring BOC actives sites. Besides, Pt nanoparticles aggregation is less favorable for photocatalysis. While noble metals loading on the surface of the catalysts should improve the migration rate of photo-generated carriers excess quantities of noble metals loading on the surface of the catalysts should improve the migration rate of photo-generated carriers excess quantities of noble metals locally can form recombination centers for e^−^'s and h^+^'s, resulting in an improvement of photocatalytic activity.^38,42,43^ Our work demonstrates again that controlling an appropriate amount of Pt is key to achieving the best photocatalytic activity of the P-BOC.

The photocatalytic performance of the 3.0 P-BOC was further evaluated by following the degradation under visible light of two other types of organic pollutant: endocrine disrupting chemical bisphenol A (BPA) and oxytetracycline (OTC) under visible light. As shown in the Fig. 7(c and d), BPA and OTC can also be decomposed under the same exposure conditions, degradation over 150 minutes reaching 93.2% and 92%, respectively.

As is well known, the stability of the photocatalysts are important during the photocatalytic reactions. To further test the performance of the P-BOC samples, recycling experiment and XPS spectra of the 3.0 P-BOC and used 3.0 P-BOC were conducted (see Fig. S2 and S3 in the ESI†). From the results of three recycle experiment, we can see that the catalyst has certain stability, the photocatalytic performance decreased a little, which may be caused by the loss of the catalyst. On the other hand, the XPS spectra show that the used samples has no obvious differences after degradation of RhB.

### 3.4 Photocatalytic mechanism

**Fig. 8:**
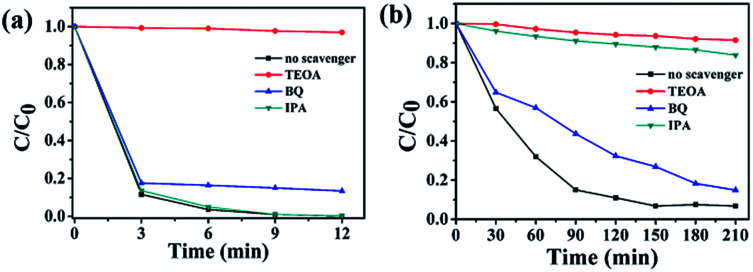
Trapping experiments of active species during the photocatalytic degradation of (a) RhB and (b) over 3.0 P-BOC under visible light irradiation.

To further explore photocatalytic performance, trapping experiments of active species, such as h^+^, ˙O_2_^−^, and ˙OH, were performed. As shown in Fig. 8(a and b), the photocatalytic activity of 3.0 P-BOC is inhibited, to varying degrees, by the addition of three types of scavengers of active species: triethanolamine (TEOA), a quencher of h^+^, and benzoquinone (BQ), a quencher of ˙O_2_^−^.^44^Fig. 8(a) shows that h^+^ and ˙O_2_^−^ both play significant roles in the degradation process of RhB. By contrast tertiary butanol (IPA, a quencher of ˙OH),^45^ has no observable delirious effect, suggesting that ˙OH is not an active species in the reaction. However, during the degradation of BPA (in Fig. 8(b)), OH also play the significant roles.

**Fig. 9:**
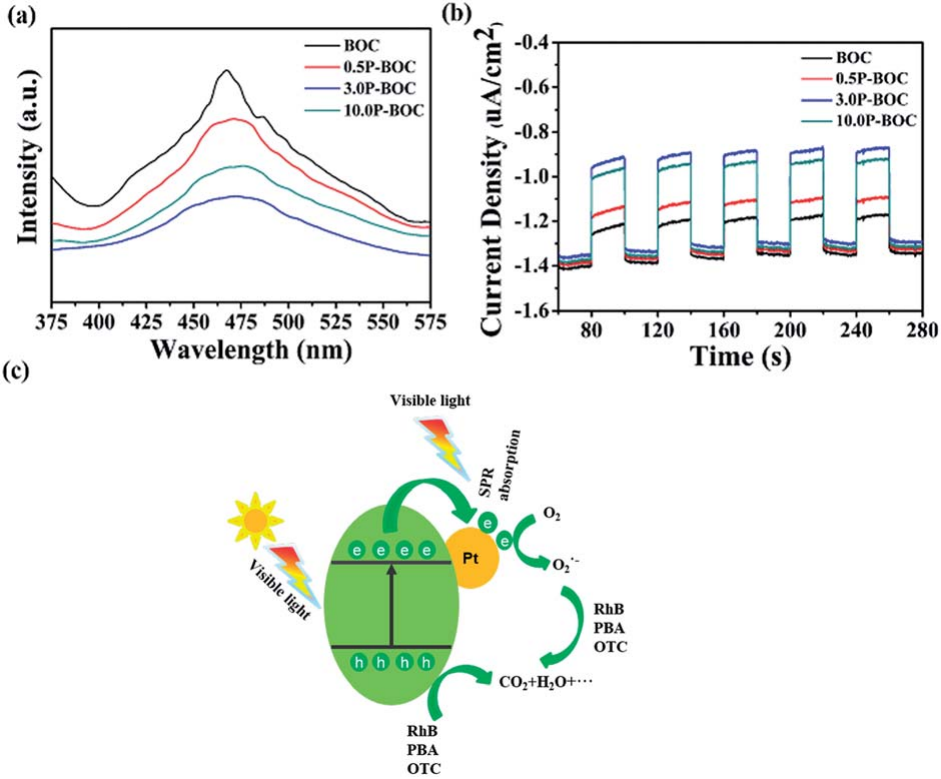
(a) PL spectra of different samples with the excitation wavelength of 300 nm. (b) The transient photocurrent responses for the BOC and the P-BOC. (c) Schematic diagram of electron–hole separation mechanism upon visible excitation for the P-BOC.

Photoluminescence (PL) was used to investigate the recombination rate of the photo-generated electrons and holes. Fig. 9(a) shows the PL spectra of the BOC and three P-BOC samples stimulated at 300 nm. All samples exhibit an emission peak at about 470 nm; the PL intensity decreases gradually and then increases with the increasing of the Pt content, consistent with the corresponding changes of photocatalytic activity. The results imply that the existence of Pt nanoparticles can inhibit the recombination of electrons and holes and furthermore improve the photocatalytic activity of the photocatalysts. Furthermore, the photocurrent responses can also supply some proof for the separation rate of electrons and holes, the transient photocurrent responses of the photocatalysts were monitored and shown in the Fig. 9(b). As illustrated in the Fig. 9(b), all samples show a rapid photocurrent and the photocurrent are steady and reproducible during several on–off cycles. Compare to the BOC, the photocurrent generated from P-BOC obvious increase, implying that the P-BOC samples have a higher separation rate of the photo-generated electrons and holes, which make the electrons have a longer lifetime. Therefore, the addition of the Pt can efficiently inhibit the recombination of electrons and holes and thus improve the photocatalytic activity.

Based on the above results, a mechanism is proposed following (Fig. 9(c)). Electrons on the valance band (VB) in BOC are photo-excited into the conduction band (CB), leaving holes behind when the photocatalyst is irradiated.^46^ Generally, photo-generated electrons and holes quickly recombine allowing only a few to participate in the photocatalytic reactions. However, when Pt nanoparticles are present, photoelectrons transfer more easily at the surface of Pt and combine with the oxygen in the solution to form ˙O_2_^−^, which is one of the active species.^47^ Besides, the holes that remain in the VB have strong adsorption effect with respect to organic pollutants causing them to decompose into small molecules, such as, H_2_O and CO_2_.^48,49^

## 4. Conclusions

In summary, a series of the P-BOC photocatalysts have been synthesized by depositing Pt nanoparticles on the surface of the rose-like Bi_2_O_2_CO_3_ and the photocatalytic performance is evaluated by decomposing RhB, BPA and OTC under visible light. In particular the photocatalytic efficiency of Bi_2_O_2_CO_3_ can be improved significantly, and the best performance is occurring for 3.0 wt% Pt. Furthermore our results show Pt nanoparticles can restrain the recombination of electrons and holes by trapping electrons, thus improving significantly the photocatalytic efficiency.

## Conflicts of interest

There are no conflicts to declare.

## Supplementary Material

RA-008-C7RA12236E-s001
