# Supplementary material for: Pt nanoparticles decorated rose-like Bi2O2CO3 configurations for efficient photocatalytic removal of water organic pollutants
Source: RSC Adv. 2018 Jan 3;8(2):914–20. doi: 10.1039/c7ra12236e (PMC9076944; doi:10.1039/c7ra12236e)
Supplement: RA-008-C7RA12236E-s001 [file RA-008-C7RA12236E-s001.pdf]

Electronic Supplementary Material (ESI) for RSC Advances.
This journal is © The Royal Society of Chemistry 2018

Pt nanoparticles decorated rose-like $Bi_2O_2CO_3$ configurations for efficient photocatalytic removal of water organic pollutants

Huijuan Chen[a], Zhongfu Zhou[b,c,d]*, G. Neville Greaves[d,e,f], Salma Nigar[a], Huaqiang Cao[g], Tingkai Zhao[h] & Xionggang Lu[b]

a. School of Material Science and Engineering, Shanghai University, Shanghai 200444, PR China
b. State Key Laboratory of Advanced Special Steel, Shanghai University, Shanghai 200072, PR China.
c. Key Laboratory of Material Microstructures, Shanghai University, Shanghai 200444, PR China.
d. Department of Physics, Aberystwyth University, Aberystwyth SY23 3BZ, UK.
e. State Key Laboratory of Silicate Materials for Architectures, Wuhan University of Technology, Wuhan 430070, China.
f. Department of Materials Science and Metallurgy, University of Cambridge, Charles Babbage Road, Cambridge, CB2 3QZ, United Kingdom.
g. Department of Chemistry, Tsinghua University, Beijing 100084, PR China.
h. State Key Laboratory of Solidification Processing, School of Materials Science and Engineering, Northwestern Polytechnical University, Xi'an 710072, PR China.

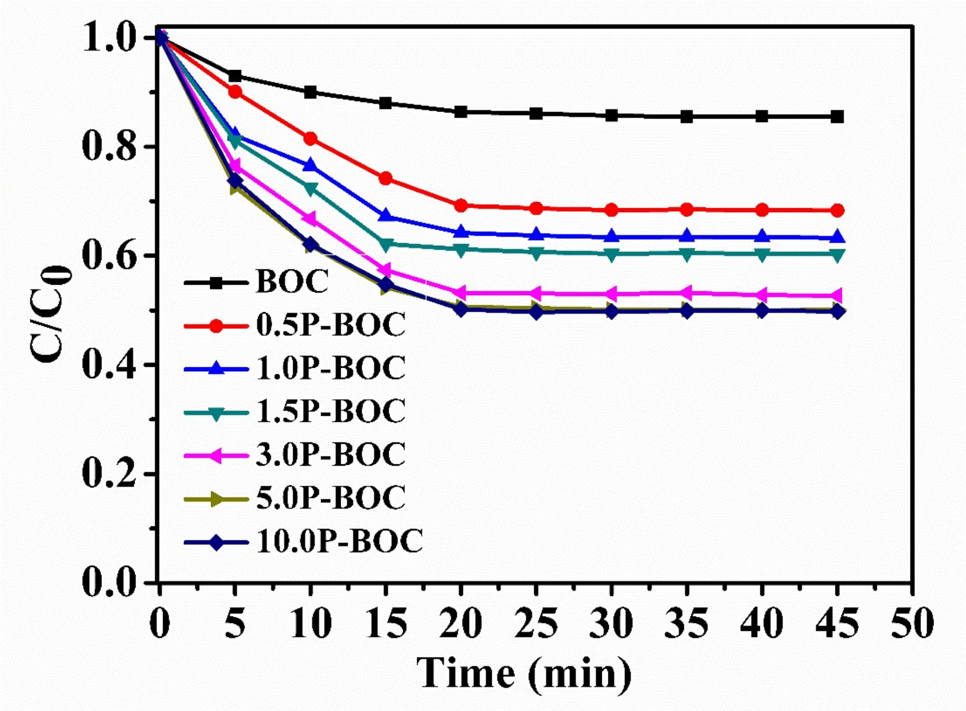

Figure S1. The adsorption-desorption equilibrium curves before light irradiation.

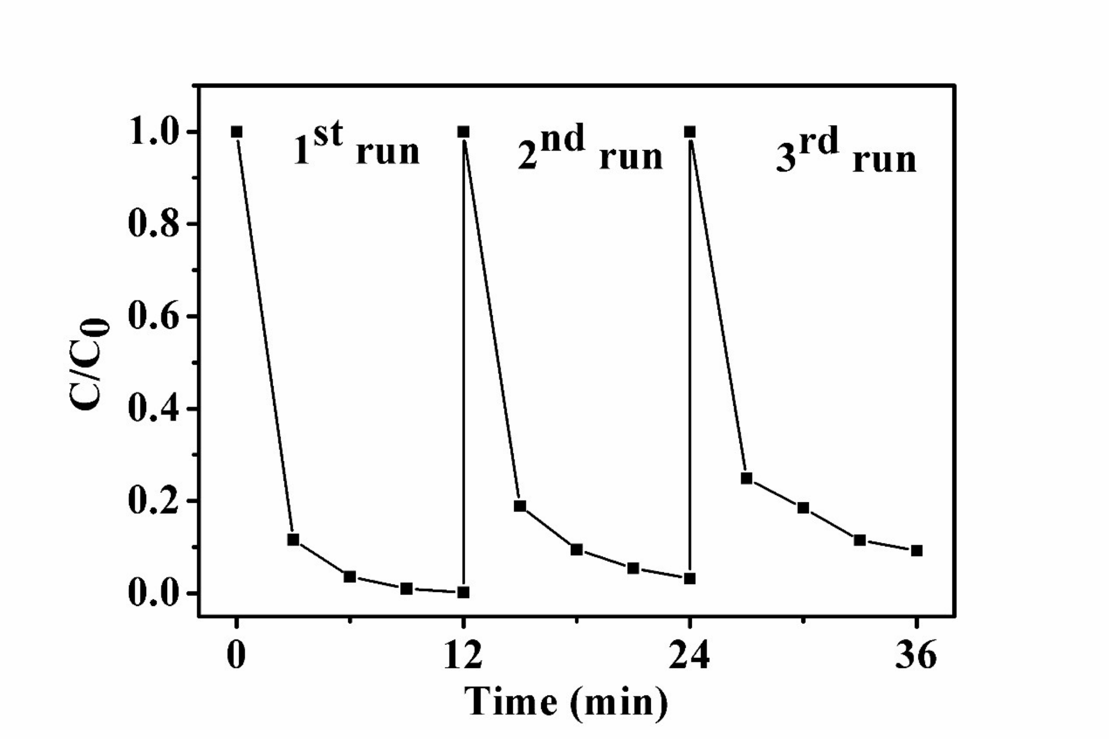

Figure S2. The cycling experiment of the 3.0 P-BOC for degradation of RhB.

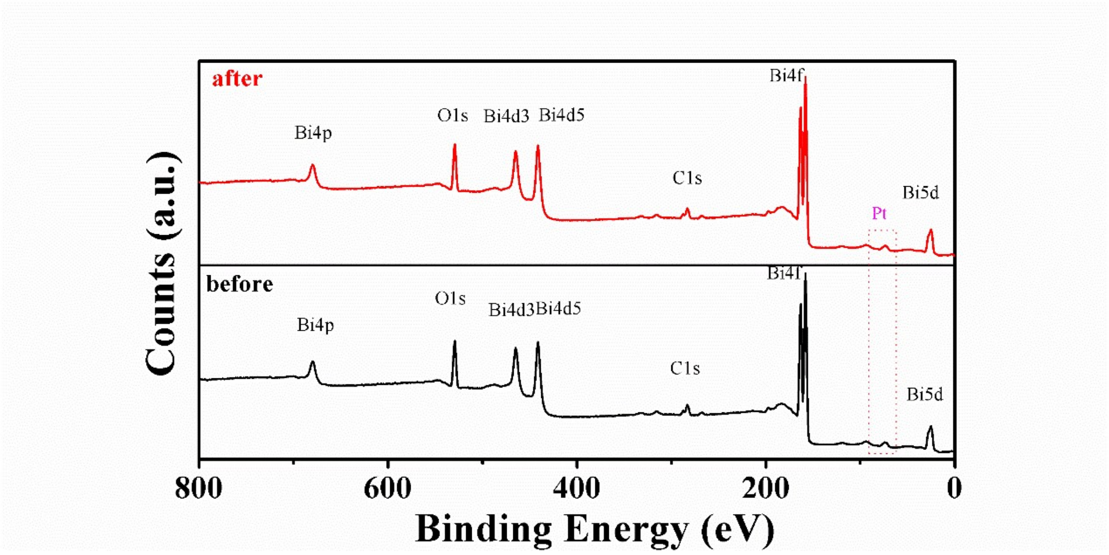

Figure S3. XPS spectra of the 3.0 P-BOC before and after degradation of RhB.
